# Carpal tunnel syndrome caused by a giant lipoma of the hand: A case report

**DOI:** 10.1016/j.ijscr.2021.105647

**Published:** 2021-02-15

**Authors:** Belle Tellier, Mariam Gabrian, Jean-Bart Jaquet

**Affiliations:** a Plastic, Reconstructive and Hand Surgery Department, Maasstad Hospital, Rotterdam, The Netherlands; b Department of General Surgery, Erasmus Medical Center, Rotterdam, The Netherlands

**Keywords:** Giant, Lipoma, Hand surgery, Carpal tunnel, Case report

## Abstract

**Introduction and importance:**

Lipomas are common benign tumours which occur in up to 2% of the population. They are classified as giant when larger than 5 cm. Although they are usually asymptomatic, giant lipomas of the hand may cause compression of the underlying tissues.

**Case presentation:**

A 62-year-old Caucasian male presented to the Plastic and Reconstructive Surgery outpatient clinic with numbness and pain in his left hand. The numbness in his fingers pointed to compression of the median nerve, as well as the ulnar nerve. He presented with a rapidly progressive swelling in his left palm. An MRI scan of the hand was made, which showed a lipoma of approximately 8,5 cm in diameter. The swelling was surgically removed and sent for histopathological analysis, which confirmed the diagnosis of benign giant lipoma of the hand. Two weeks postoperatively, pain and numbness significantly decreased.

**Clinical discussion:**

Neural injury in carpal tunnel syndrome is related to the duration and degree of compression. A giant lipoma is considered malignant until proven otherwise since variants with high potential for metastasizing exist. Distinguishing between a benign tumour and a malignant lipoma is essential, since a more radical treatment plan might be required.

**Conclusion:**

Giant lipomas of the hand are a rare cause of carpal tunnel syndrome and a malignant variant should always be suspected. A preoperative MRI scan should be performed. Rapid en bloc excision is necessary in case of compression of the underlying tissues.

## Highlights

• Giant lipomas of the hand are a rare cause of carpal tunnel syndrome.
• A preoperative MRI scan should be performed.
• Rapid en bloc excision is necessary in case of compression of underlying tissues.

## 1 Introduction and importance

A lipoma is a common subcutaneous mesenchymal benign tumour, which occurs in 2% of the population. Localization in distal extremities is uncommon, occurring in less than 1% of cases [1,2]. It is classified as giant if it exceeds 5,0 cm [[1], [2], [3], [4]]. A giant lipoma in the hand is usually asymptomatic. In 25% of cases it can cause compression of surrounding tissues [1,2].

Direct compression of the median or ulnar nerve can result in symptoms such as paraesthesia, pain and loss of strength [1,2]. In case of median nerve neuropathies, which account for 90% of entrapment neuropathies, paraesthesia is seen in the first three digits and radial half of the fourth digit; night-time pain and a positive flick sign are other commonly seen symptoms [5]. Risk factors associated with carpal tunnel syndrome (CTS) are vibration exposure and wrist position. Patients with diabetes, obesity, alcoholism, thyroid disease and rheumatoid arthritis have an increased likelihood of developing CTS [6]. When a growing mass exerts pressure on a nerve, the blood nerve barrier is affected resulting in perineural edema and fibrosis. In cases of chronic compression, demyelination followed by degeneration occur. Cases with a low degree of neural injury are more likely to show remyelination and in time recover function [6]. Although it is an atypical cause of CTS, some cases of median nerve entrapment triggered by a giant lipoma have been reported [1,4].

While most lipomas are benign, a rapidly growing and painful mass is more likely to be malignant [1]. A giant lipoma is considered malignant until proven otherwise [7], since variants with high potential for metastasizing exist [8,9]. Magnetic resonance imaging (MRI) is recommended to determine the presence of malignant characteristics. Furthermore, it also allows detailed visualization of the mass in relation to other viable structures [1,4,8]. This type of examination has a 94% diagnosis rate in detecting masses in the hand and wrist [4]. Biopsy of the mass should be considered in case of malignant features preoperatively [2]. Only histopathological examination can confirm a definitive diagnosis of a benign giant lipoma [3].

## 2 Case presentation

The work has been reported in line with the SCARE 2020 criteria [10]. A 62-year-old male presented to the Plastic and Reconstructive Surgery outpatient clinic with a growing mass in his left palm, which suddenly appeared 18 years ago and had not increased in size before. However, it recently started to grow, causing progressive symptoms of pain and loss of sensory and motor function of both the median and ulnar nerve. Clinically, the mass was localized in the hand palm, ulnar from the thenar eminence and measured approximately 4 × 4 cm. No relevant diseases or medication use were reported. The patient did not have a history of smoking or drug/alcohol abuse.

**Fig. 1:**
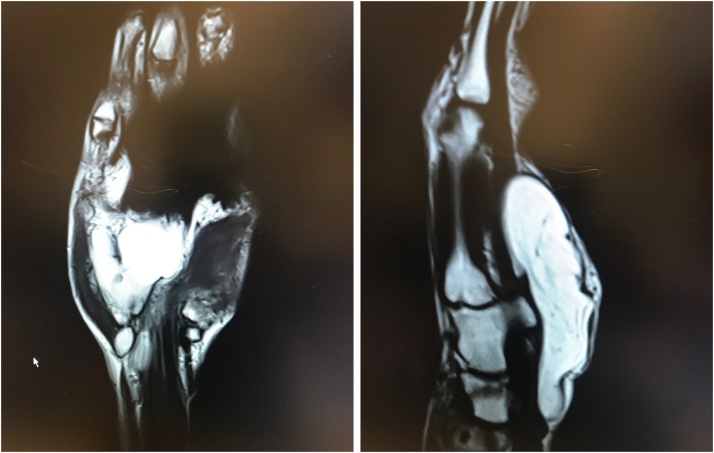
MRI-scan of the hand depicting the fatty tissue tumour. The mass was localized ventrally from the flexor tendons, measuring 4,8 × 1,8 cm in the transverse plane. In the sagittal plane, the maximum diameter was 8,6 cm.

An ultrasound showed two masses of similar density and architecture. The ulnar and radial swelling measured 27 × 14 mm and 35 × 15 mm, respectively. An MRI scan was performed which showed a lipoma of 4,8 × 1,8 × 8,6 cm volar to the flexor tendons (Fig. 1). The tumour spread out to the carpal tunnel and Guyon’s canal. A hypo-intense fibrous band was seen, which could explain the illusion of two separate masses on ultrasound. Some oedema was present, but there was no suspicion of a malignant tumour.

Due to the progression of symptoms, rapid excision of the mass was recommended. The carpal tunnel was opened through an l-shaped skin incision in the palmar crease. Secondly, the median nerve was identified and protected after dissection of the superficial component of the lipoma. Subsequently, we proceeded to dissect the deep palmar space and the component located in Guyon’s canal. The branch of the ulnar nerve in Guyon’s canal was spared.

**Fig. 2:**
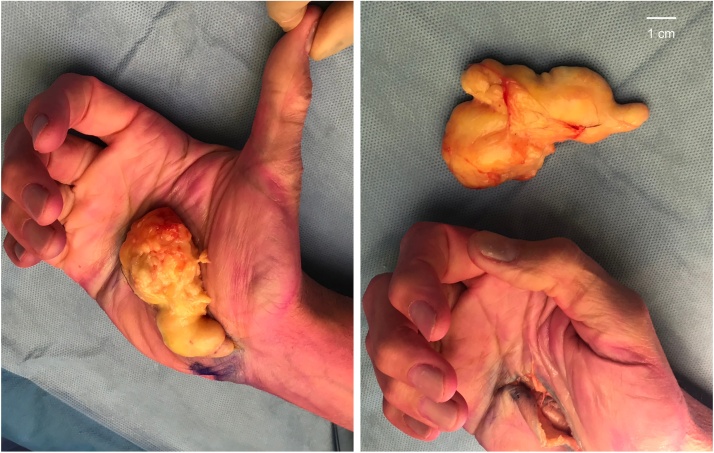
Total excision of the giant lipoma of the hand, measuring 4,8 × 1,8 × 8,6 cm. The carpal tunnel and Guyon’s canal were released to relieve pressure on the median and ulnar nerves.

The lipoma was removed completely (Fig. 2) with no postoperative complications. Pathological examination confirmed the diagnosis of a benign giant lipoma. After the operation, the patient was advised to elevate the hand for the first week to prevent swelling. Furthermore, a pressure bandage was used, which was removed after 12 days. Two weeks postoperatively, pain and numbness significantly decreased. Three months later, the patient experienced a full recovery of function of the hand.

## 3 Clinical discussion

Neural injury is related to the duration and degree of compression [6]. A giant lipoma exerting pressure on the median nerve and causing compression can eventually lead to nerve degeneration. Recovery of function is less likely in cases of chronic compression. Therefore, early removal of the mass is critical.

A giant lipoma is considered malignant until proven otherwise [7], since variants with high potential for metastasizing exist [8,9]. Therefore, in cases such as this, distinguishing between a benign lipoma and a liposarcoma is essential, as more radical treatment might be required. Liposarcomas account for approximately 20% of all soft tissue sarcomas [11]. Presence in children is very rare and these tumours seldom arise from preexisting lipomas or subcutaneous fat [3]. The well-differentiated liposarcoma (WDLS) is the largest subgroup, accounting for 40% of the cases. [8,9] WDLS and atypical lipomatous tumours (ALT) are locally aggressive with no potential of metastasizing. ALT arises more frequently in extremities, and thus is more likely in case of a malignant giant lipoma of the hand. It is often intramuscular and does not typically invade bone. WDLS and ALT share identical histological features [9].

Other more aggressive, but less common, forms with high potential for metastasizing (17–30%) are the dedifferentiated liposarcoma (DDLS); the myxoid round cell liposarcoma (MRCL); and pleiomorphic liposarcoma (PMLS). These types have high recurrence rates ranging from 34% to 45%. Interestingly, the DDLS can arise de novo or be present in recurrence of WDLS. DDLS is more often found in the retroperitoneum [3,9]. For these lesions, excision with a wide margin and adjuvant radiotherapy are desired. Complete resection diminishes the risk of recurrence [9]. For MRCL and PMLS resection of muscle groups is often necessary. A liposarcoma in the extremity without distant metastases requires limb sparing surgery combined with additional therapy in most cases [11]. In case of limb sparing surgery, adjuvant radiotherapy has shown improvement of local control. However, it has no expected survival benefit in the ALT/WDLS group since they do not metastasize [9].

## 4 Conclusion

In case of a giant lipoma of the hand, a preoperative MRI scan is highly recommended, considering the risk of malignant potential. Furthermore, detailed visualization of the mass in relation to other viable structures is an asset [1,4,8]. We believe rapid en bloc excision of a giant lipoma is necessary in case of compression. For every patient, an individualized trajectory is needed with diligent planning. Furthermore, we believe good postoperative results can be achieved, without permanent damage to the viable structures involved.

## Declaration of competing interest

Nothing to declare.

## Funding

Nothing to declare.

## Ethical approval

No ethical approval was necessary.

## Consent

Written informed consent was obtained from the patient for publication of this case report and accompanying images. A copy of the written consent is available for review by the Editor-in-Chief of this journal on request.

## Author contribution

All authors contributed to the conception and design. Jean-Bart Jaquet and Mariam Gabrian performed the surgery. All authors contributed to drafting, critical revision, and final approval of the article.

## Registration of research studies

Not Applicable.

## Guarantor

Jean-Bart Jaquet, Belle Tellier and Mariam Gabrian are all guarantors for the report.

## Provenance and peer review

Not commissioned, externally peer-reviewed.
